# How machine learning could be used in clinical practice during an epidemic

**DOI:** 10.1186/s13054-020-02962-y

**Published:** 2020-05-26

**Authors:** Charles Verdonk, Franck Verdonk, Gérard Dreyfus

**Affiliations:** 1 Department of Neurosciences and Cognitive Sciences, Unit of Neurophysiology of Stress, French Armed Forces Biomedical Research Institute, 91220 Brétigny-sur-Orge, France; 2 ESPCI Paris – PSL University, 75005 Paris, France; 3 Department of Anaesthesiology, Perioperative and Pain Medicine, Stanford University School of Medicine, Stanford, CA 94305 USA; 4 Department of Anaesthesiology and Intensive Care, Hôpital Saint-Antoine, Assistance Publique-Hôpitaux de Paris, 75012 Paris, France

The COVID-19 epidemic is the cause of a crisis that is confronting the healthcare community with an unprecedented situation: emergency and intensive care units (ICU) are saturated, compelling physicians to make extremely hard decisions (triage). In such a resource-constrained situation, physicians need decision support systems that could help them to optimally stratify patient risk. In the present paper, we explain how machine learning (ML) could help clinical practitioners during the epidemic (Fig. 1), and we describe some challenges and perspectives that could direct future efforts in the field.

**Fig. 1:**
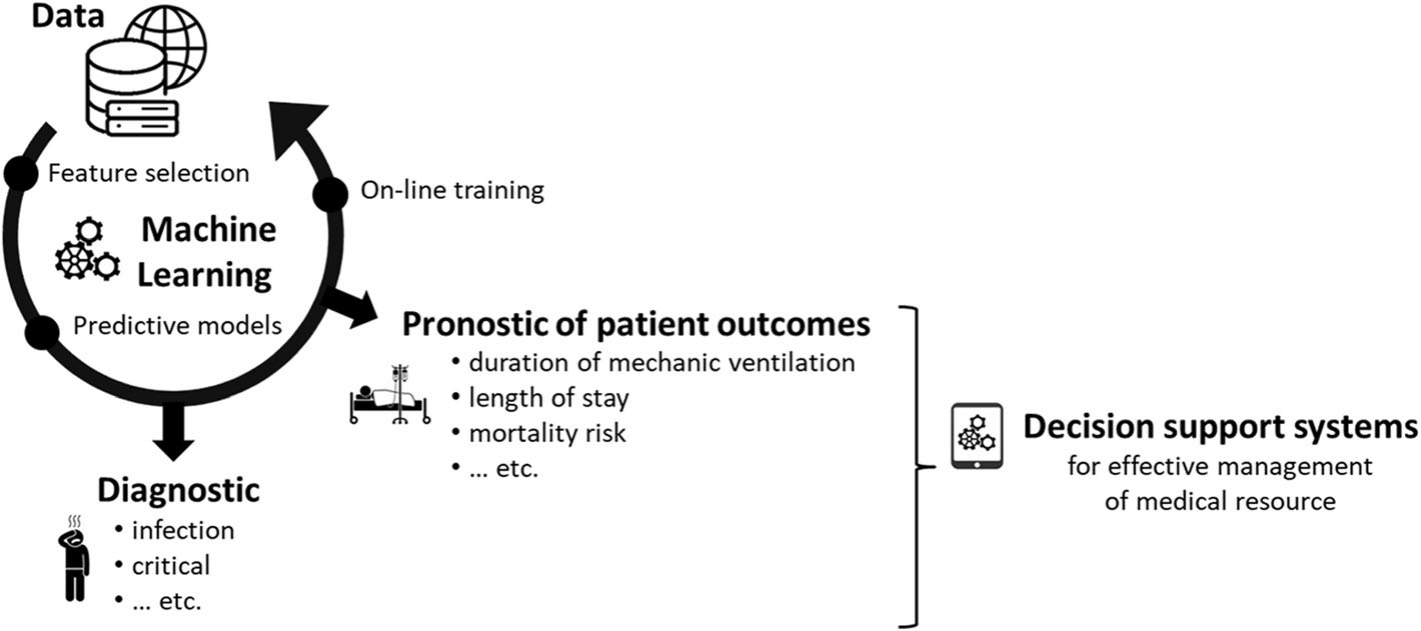
Machine learning-based decision support systems can help clinical practice during an epidemic. Efficient diagnostic and accurate prediction of patient outcomes can ultimately lead to effective medical resource management. In contrast to traditional approaches, machine learning algorithms enable feature selection and design of non-linear models that improve prediction of clinical outcomes, and on-line training techniques allow upgrading of decision support systems, as the data regarding the epidemic increases

## Why might machine learning be of particular interest?

The first point to note is that ML encompasses a wide range of data analysis algorithms that run along a continuum from partially to fully machine-guided [1, 2]. For instance, deep learning is a fully machine-guided approach that uses large combinations of mathematical functions called “artificial neurons”. Although recent contributions are promising [3], deep learning is not seen as particularly useful by some of the healthcare community because it produces black box models that are not readily interpretable by physicians [4]. Therefore, efforts to develop ML-based decision support systems for clinical practice should focus on models that incorporate as much prior medical knowledge as possible.

A controversial step in traditional decision-making algorithms is the selection of variables (often termed “features”) that are included in the predictive model. Classically, experts in the field work together to select features that they believe to be relevant to the research question. Feature selection is essential in model design, because the inclusion of irrelevant features is detrimental to the understanding and performance of the model, while rejecting relevant features can be just as bad [5]. Recent times have seen the emergence of “big” healthcare data that increase the number of candidate features. Here, we argue that feature selection should be performed by ML algorithms, rather than humans.

A reductionist view assumes that the prediction of patient outcomes relies on the sum of risk factors, as is the case with most scoring systems. We believe that such a reductionist approach is limited in its ability to successfully predict patient outcomes. The majority of health conditions (e.g., asymptomatic, infection, or critical) do not arise from a linear interaction between isolated factors, but from non-linear interactions among a web of determinants (genetic, biological, clinical, social, etc.). Neural networks, a family of ML nonlinear models (of which deep learning is a variant), are a particularly attractive way to model this, not least because of their universal approximation property [6].

At the same time, an epidemic context is characterized by a high risk of a shortage of medical equipment (e.g., ventilators). Therefore, there is a critical need to be able to predict resource consumption (e.g., the duration of mechanical ventilation) and patient outcomes (e.g., mortality risk). Neural networks can be used in regression and classification tasks as a function of the type of outcome (quantitative or categorical) to be predicted [6].

Finally, the time course of an epidemic is characterized by a gradual increase in information regarding the disease, as the number of infected patients increases. It should be possible to train decision support systems with dynamic datasets that grow as the amount of data increases; to this end, “stochastic” or “on-line” training techniques are available. Another key issue, in the context of an epidemic, is the choice of “informative” data. The latter needs to be carefully selected, in order to avoid choking training algorithms with redundant information. This problem can be addressed by active learning methods.

## A challenge for machine learning in healthcare: linking biomedical data

Despite the many fascinating applications of ML in healthcare [2, 7], some challenges remain to be overcome before it can be deployed as a support to clinical practice during an epidemic. First and foremost, ML algorithms need to have access to sufficient amounts of data that closely resemble the data expected in a clinical scenario. Therefore, we encourage the healthcare community and organizations to make biomedical data public as early as possible and right from the beginning of the epidemic. The COVID-19 epidemic promotes the constitution of medical databases (e.g., COVID ICU, French corona) that will be exhaustive enough to allow ML, but they are currently not public.

Once the data are made available, the next challenge is to aggregate disparate datasets that are scattered across hospitals. Processing big healthcare data requires a variety of skillsets; this can only be provided by multidisciplinary teams that include physicians and ML experts. If this does not happen, predictive models are likely to be irrelevant, due to data misrepresentation. Consequently, the integration of ML-based decision support systems into clinical practice becomes hopeless.

## An early illustration of ML-based decision support systems

A search of the PubMed database (see Supplementary material) highlights some promising proofs of concept regarding the ability of ML-based technologies to assist medical decision-making during an epidemic.

For instance, Yao et al. [8] have developed a screening system that can detect individuals infected with influenza from three clinical features (heart rate, respiration rate, and facial temperature). Their system is especially interesting as it uses contactless technologies that make it particularly suitable for clinical application with contagious patients [8]. More recently, Dagdanpurev et al. [9] have developed a similar screening system using the same three clinical features. Their system uses a random tree algorithm to predict the patient’s infection status and is easily understood by physicians because it can be expressed as a flow chart [9].

Another promising ML-based decision support system is a mobile app that provides physicians with access to clinical guidelines. Colubri et al. [10, 11] designed ML-based models that can predict the mortality risk of patients infected with Ebola. Their models were subsequently incorporated into a mobile app to support medical decision-making in isolated clinical care settings [10, 11]. These systems are particularly attractive during an epidemic because they can be readily deployed and regularly updated as new biomedical information becomes available.

## Supplementary information

**Additional file 1.**

## Data Availability

Not applicable
