# Supplementary material for: How machine learning could be used in clinical practice during an epidemic
Source: Crit Care. 2020 May 26;24:265. doi: 10.1186/s13054-020-02962-y (PMC7250254; doi:10.1186/s13054-020-02962-y)
Supplement: Supplementary file 1 — Additional file 1. [file 13054_2020_2962_MOESM1_ESM.docx]

**Supplementary material**

This supplementary material summarises our search strategy and the results of the rapid review mentioned in the paper.

**Search strategy**

- Database: Pubmed
- Search strings
  1. COVID-19.tw^[[1]](#footnote-1)^. *OR* Ebola.tw. *OR* SARS^[[2]](#footnote-2)^.tw. *OR* influenza.tw. *OR* MERS^[[3]](#footnote-3)^.tw. *AND* machine learning
  2. COVID-19.tw. *OR* Ebola.tw. *OR* SARS.tw. *OR* influenza.tw. *OR* MERS.tw. *AND* predictive model
  3. COVID-19.tw. *OR* Ebola.tw. *OR* SARS.tw. *OR* influenza.tw. *OR* MERS.tw. *AND* prognostic model
- only articles published before 23 March 2020 were considered


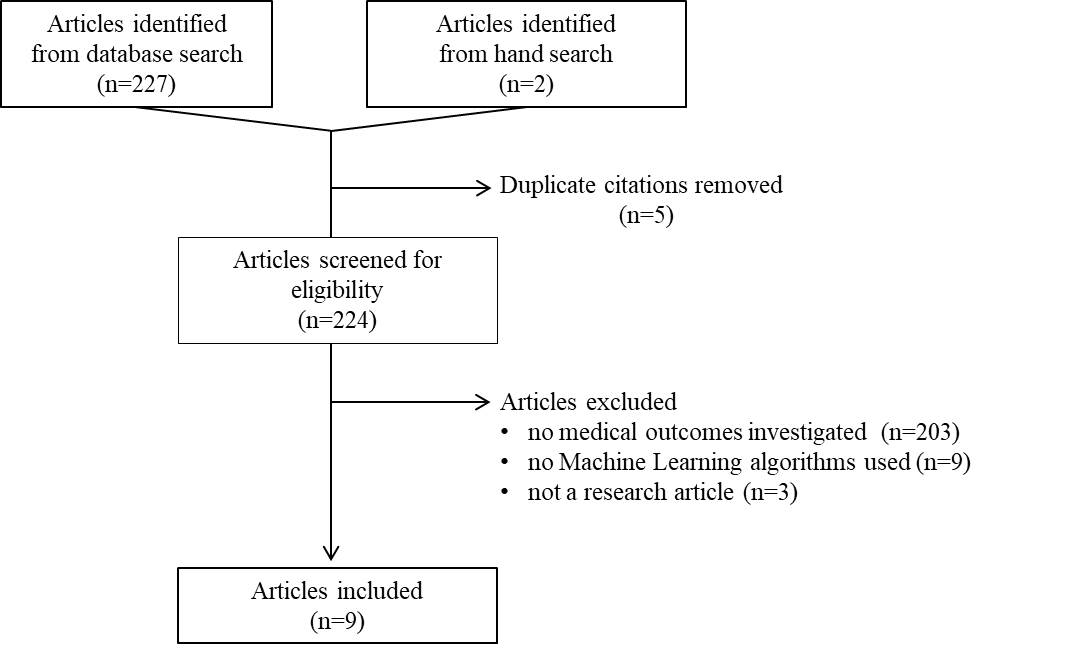


Figure 1 - Flow diagram showing articles included in the review.

**Results**

Table 1 - Characteristics of Machine Learning studies that have attempted to predict outcomes of epidemics using biomedical data.

| Authors | Epidemic | Variables | | Method | | Observations |
| --- | --- | --- | --- | --- | --- | --- |
|  |  | Features | Response | Approach | Model |  |
| John (2019) | MERS | 🗸demographic  🗸clinical | recovery | classification | SVM^[[4]](#footnote-4)^, Bayesian classifier, conditional inference tree, and J48 | Age, pre-existing disease, status at time of identification of disease (critical), and healthcare worker status (No) are associated with lower recovery |
| Price (2020) | Ebola | 🗸genetic | survival | classification | Random forest | Some gene expression programs (initially identified on the basis of a mouse model) predict survival in human patients |
| Forna (2019) | Ebola | 🗸demographic  🗸clinical | Case Fatality Ratio^[[5]](#footnote-5)^ | classification | Boosted regression tree model | Demographic features (district, case classification and quarter), hospitalization status and age predict the Case Fatality Ratio |
| Colubri (2019) | Ebola | 🗸clinical  🗸biology | survival | selection of variables | Elastic Net regularization | Clinical (age, temperature, bleeding, jaundice, dyspnoea, dysphagia and time-to-presentation) and biology (viral load) predict survival |
| Colubri (2016) | Ebola | 🗸clinical  🗸biology | survival | selection of variables, classification | Neural networks, SVM, decision tree | Biology (viral load, liver enzymes, creatinine and total CO_2_) and clinical (heart rate, diarrhoea, weakness and vomit) variables predict survival |
| Hu (2020) | Influenza | 🗸clinical  🗸biology | survival | classification | Gradient boosting machine, random forest | Intensive care unit specific indices (e.g. PaO_2_/FiO_2_, APACHE II score, etc.), biology and clinical variables predict survival |
| Yao (2016) | Influenza | 🗸clinical | infection status | classification | LDA^[[6]](#footnote-6)^, QDA^[[7]](#footnote-7)^, SVM, Bayesian classifier, k-NN^[[8]](#footnote-8)^ | Heart rate, respiration rate, and facial temperature predict infection status |
| Dagdanpurev (2019) | Influenza | 🗸clinical | infection status | classification | Random tree algorithm | Heart rate, respiratory rate, and axillary temperature predict infection status |
| Patel (2018) | Asthma exacerbation | 🗸clinical  🗸demographic | hospitalization | classification | Decision trees, random forests and gradient boosting machines | Patient vital signs (SatO_2_, respiratory rate, and heart rate), triage acuity (Emergency Severity Index), age, weight, socioeconomic status and weather-related features predict hospitalization. |

**References**

1. John M, Shaiba H. Main factors influencing recovery in MERS Co-V patients using machine learning. J Infect Public Health. 2019;12(5):700-4.

2. Price A, Okumura A, Haddock E, Feldmann F, Meade-White K, Sharma P, et al. Transcriptional Correlates of Tolerance and Lethality in Mice Predict Ebola Virus Disease Patient Outcomes. Cell Rep. 2020;30(6):1702-13.

3. Forna A, Nouvellet P, Dorigatti I, Donnelly CA. Case fatality ratio estimates for the 2013–2016 West African Ebola epidemic: application of Boosted Regression Trees for imputation. Int J Infect Dis. 2019;79:128-35.

4. Colubri A, Hartley MA, Siakor M, Wolfman V, Felix A, Sesay T, et al. Machine-learning Prognostic Models from the 2014–16 Ebola Outbreak: Data-harmonization Challenges, Validation Strategies, and mHealth Applications. EClinicalMedicine. 2019;11:54-64.

5. Colubri A, Silver T, Fradet T, Retzepi K, Fry B, Sabeti P. Transforming clinical data into actionable prognosis models: machine-learning framework and field-deployable app to predict outcome of Ebola patients. PLoS Negl Trop Dis. 2016;10(3):e0004549.

6. Hu CA, Chen CM, Fang YC, Liang SJ, Wang HC, Fang WF, et al. Using a machine learning approach to predict mortality in critically ill influenza patients: a cross-sectional retrospective multicentre study in Taiwan. BMJ Open. 2020;10(2):e033898.

7. Yao Y, Sun G, Matsui T, Hakozaki Y, van Waasen S, Schiek M. Multiple vital-sign-based infection screening outperforms thermography independent of the classification algorithm. IEEE Trans Biomed Eng. 2016;63(5):1025-33.

8. Dagdanpurev S, Abe S, Sun G, Nishimura H, Choimaa L, Hakozaki Y, et al. A novel machine-learning-based infection screening system via 2013–2017 seasonal influenza patients' vital signs as training datasets. J Infection. 2019;78(5):409-21.

9. Patel SJ, Chamberlain DB, Chamberlain JM. A Machine Learning Approach to Predicting Need for Hospitalization for Pediatric Asthma Exacerbation at the Time of Emergency Department Triage. Acad Emerg Med. 2018;25(12):1463-70.

1. Title and Abstract fields were searched [↑](#footnote-ref-1)
2. SARS: Severe Acute Respiratory Syndrome [↑](#footnote-ref-2)
3. MERS: Middle East Respiratory Syndrome [↑](#footnote-ref-3)
4. Support Vector Machine [↑](#footnote-ref-4)
5. Case fatality ratio: proportion of deaths compared to the total number of people diagnosed with the disease over a certain period of time [↑](#footnote-ref-5)
6. LDA: Linear Discriminant Analysis [↑](#footnote-ref-6)
7. QDA: Quadratic Discriminant Analysis [↑](#footnote-ref-7)
8. k-NN: k-Nearest Neighbours [↑](#footnote-ref-8)
